# Extracellular Vesicles Can Deliver Anti-inflammatory and Anti-scarring Activities of Mesenchymal Stromal Cells After Spinal Cord Injury

**DOI:** 10.3389/fneur.2019.01225

**Published:** 2019-11-29

**Authors:** Pasquale Romanelli, Lara Bieler, Cornelia Scharler, Karin Pachler, Christina Kreutzer, Pia Zaunmair, Dominika Jakubecova, Heike Mrowetz, Bruno Benedetti, Francisco J. Rivera, Ludwig Aigner, Eva Rohde, Mario Gimona, Dirk Strunk, Sebastien Couillard-Despres

**Affiliations:** ^1^Institute of Experimental Neuroregeneration, Paracelsus Medical University, Salzburg, Austria; ^2^Spinal Cord Injury and Tissue Regeneration Center Salzburg (SCI-TReCS), Salzburg, Austria; ^3^Institute of Experimental and Clinical Cell Therapy, Paracelsus Medical University, Salzburg, Austria; ^4^GMP Laboratory, Paracelsus Medical University, Salzburg, Austria; ^5^Institute of Molecular Regenerative Medicine, Paracelsus Medical University, Salzburg, Austria; ^6^Laboratory of Stem Cells and Neuroregeneration, Faculty of Medicine, Institute of Anatomy, Histology and Pathology, Universidad Austral de Chile, Valdivia, Chile; ^7^Center for Interdisciplinary Studies on the Nervous System (CISNe), Universidad Austral de Chile, Valdivia, Chile; ^8^Austrian Cluster for Tissue Regeneration, Vienna, Austria; ^9^University Department of Transfusion Medicine, University Clinic Salzburg Paracelsus Medical University, Salzburg, Austria

**Keywords:** exosome, inflammation, mesenchymal stromal cells, scarring, spinal cord injury

## Abstract

Spinal cord injury is characterized by initial neural tissue disruption that triggers secondary damage and extensive non-resolving inflammation, which aggravates loss of function and hinders recovery. The early onset of inflammation following traumatic spinal cord injury underscores the importance of acute intervention after the initial trauma. Injections of mesenchymal stromal cells (MSCs) can reduce inflammation following spinal cord injury. We asked if extracellular vesicles (EVs) can substitute the anti-inflammatory and anti-scarring activities of their parental MSCs in a rat model of contusion spinal cord injury. We report that MSC-EVs were as potent as the parental intact cells in reducing the level of neuroinflammation for up to 2 weeks post-injury. Acute application of EVs after spinal cord injury was shown to robustly decrease the expression of pro-inflammatory cytokines in the spinal cord parenchyma in the very early phase of secondary damage. Moreover, the anti-scarring impact of MSC-EVs was even more efficient than the parental cells. We therefore conclude that anti-inflammatory and anti-scarring activities of MSC application can be mediated by their secreted EVs. In light of their substantial safety and druggability advantages, EVs may have a high potential in early therapeutic treatment following traumatic spinal cord injury.

## Introduction

Traumatic spinal cord injury (tSCI) results from the sudden and largely irreversible disruption of neural parenchyma by mechanical forces, damaging axons and neuronal cell bodies, glia, and blood vessels. The latter is results in the formation of edema and ischemia. Importantly, this initial tissue disruption triggers processes of secondary damage within minutes, and these are characterized by massive inflammation and cause further tissue loss (1–3). At the lesion site, the invasion of leukocytes results in the rapid accumulation of pro-inflammatory and pro-apoptotic molecules, such as interleukin-1β (IL-1β) and tumor necrosis factor α (TNFα), as well as lytic enzymes (4). Moreover, the persistence of immune cells at the lesion site following tSCI sustains a toxic neuroinflammation that amplifies tissue loss and hinders functional recovery (5–7). Although the acute phase of inflammation is generally associated with deleterious impact, the later or chronic phases are more often supportive of regeneration (3). In contrast to other types of central nervous system (CNS) lesions, the inflammation following tSCI fails to convert into a regenerative type as well as to eventually resolve (5, 7). This harmful inflammation in cases of tSCI underscores the importance of controlling the inflammatory processes as early as possible after initial trauma.

Correlations between poor prognosis and the accumulation of pro-inflammatory cytokines, such as IL-1β, IL-6, and TNFα, and a better functional outcome with increased levels of anti-inflammatory factors, such as IL-10, have been described in tSCI patients (8, 9). Thus, the composition of the pro- and anti-inflammatory milieu and the degree of leukocyte recruitment at the lesion site are highly relevant to a functional outcome following tSCI and constitute a critical therapeutic target. Earlier studies indicate that the application of mesenchymal stromal cells (MSCs) can promote a protective and even regenerative environment after tSCI. For example, the systemic administration of umbilical cord blood MSCs have been shown to improve hind limb function in a rat tSCI model and was associated with increased serum levels of IL-10 and decreased the levels of TNFα (10).

Intravenous application of MSCs resulted in a significant reduction of inflammation, despite being rapidly cleared from the circulation and accumulating in the lung, liver, and spleen (11, 12). MSCs appear capable of modulating inflammation over distance via secreted factors rather than engrafting at the site of injury (13). In recent years, extracellular vesicles (EVs) have been recognized as paracrine factors that are released by almost all cell types (14, 15). EVs form a heterogeneous family of extracellular nanovesicles comprising exosomes, microvesicles, and apoptotic bodies (16). EVs contain numerous bioactive molecules as cargo, including mRNAs, miRNAs, and growth factors (17). MSC-EVs can recapitulate some of the anti-inflammatory activities of MSCs, such as stimulating the production of IL-10 and decreasing the expression of TNFα and IL-1β in rat models of nerve injury (18, 19).

It remains to be determined to which extent the EV activities compare to those of the parental cells in terms of pleiotropism and potency. Despite the reported benefits in various animal models of tSCI, the mode of action and therapeutic potential of EVs compared to live MSCs remains to be elucidated (20). In this work, we compared in a rat model of tSCI the impact of very early intravenous application of either human umbilical cord MSC-derived EVs (hUC-MSC-EVs) or their parental hUC-MSCs on secondary damage processes, including inflammation, scarring, and further parenchymal loss.

## Materials and Methods

### Preparation of hUC-MSC

Human umbilical cord (hUC)-derived MSCs from five donors were isolated as previously described (21). Approval was obtained for human cell and tissue sample collection from the Institutional Review Board (protocol 415-E/1776/4-2014, Ethics Committee of the province of Salzburg). Briefly, hUCs were obtained immediately after delivery and stored in phosphate buffered saline (PBS) containing heparin (10 IU/mL, Biochrome AG, Germany) until further processing. Whole cords were washed three times with PBS to remove contaminating blood cells before the hUC stroma (containing Wharton's jelly) was cut into small pieces of 1–2 mm^3^ using a sterile scalpel (sparing the cord membrane and the cord vessels). Pieces were transferred into a new culture plate to allow them to dry-adhere to the plastic surface for at least 5 min before carefully adding alpha-modified minimum essential medium (α-MEM, Sigma-Aldrich) supplemented with 10% v/v pooled human platelet lysate (pHPL), dipeptiven (1.2 mg/ml, Fresenius-Kabi), and preservative-free heparin (2 U/mL, Biochrom AG). Pooled HPL was prepared as previously described (22, 23). After 10–12 days, outgrowing MSC colonies became visible and the tissue pieces were removed gently with sterile forceps. hUC-derived MSCs were detached enzymatically and further passaged. In this study, hUC-MSCs derived from five different human donors were mixed in equal amounts prior to large-scale expansion in cell factory systems (CF4, Thermo Scientific) and used before passage five.

### Culture of hUC-MSCs and Purification of EVs

Five individual hUC-MSC lines were co-cultured in a four-layered cell factory vessel as described above. At 40% confluence, cells were washed twice with phosphate-buffered saline (PBS) and the culture medium was replaced with pHPL-EV-depleted medium, which was prepared as described (24). After 48 h, EVs were isolated from conditioned medium of the hUC-MSC pool by ultracentrifugation and filtration. Cell debris and large vesicles were removed from conditioned medium by centrifugation at 2,500 × g for 20 min and 4°C. The depletion of large microvesicles was performed by ultracentrifugation at 30,000 × g for 20 min and 18°C (ultracentrifuge WX-80, fiberlite fixed-angle rotor F37L-8 × 100, k-factor 168, Thermo Scientific). The resulting supernatant was filtered through a 0.22 μm filter and centrifuged at 120,000 × g for 180 min at 18°C to pellet hUC-MSC-EVs. EV-enriched pellets were resuspended in PBS and again collected by ultracentrifugation at 120,000 × g for 180 min at 18°C. The EV-containing pellets were dissolved in Ringer's lactate solution (0760161/02A, Fresenius Kabi) and sterile-filtered through a 0.22 μm filter. The volume of Ringer's lactate solution for EV resuspension depended on the parental cell count and was adjusted to yield 1 mL per 1 × 10^8^ parental cells. EV preparations were stored at −80°C until use. The efficiency recovery was estimated to be 10% of the starting EV amount present in the culture supernatants.

After collection of conditioned medium, the EV donor cells were detached from culture vessels by the addition of TrypLE Select CTS (A12859-01, Gibco), stained with trypan blue, and counted using a hemocytometer to determine cell number and viability. Cell aliquots were stored in medium containing α-MEM and supplemented with 20% human serum albumin (Octapharma Albunorm Infusion solution 200 g/l) and 10% DMSO (CryoSure, WAK Chemie) in liquid nitrogen until use. Prior to injection, MSCs were rescue cultured for 48–72 h. Cells were detached and harvested from culture vessels by the addition of TrypLE Select CTS (A12859-01, Gibco). hUC-MSCs were resuspended in Ringer's lactate solution (0760161/02A, Fresenius Kabi).

### Animal Groups

Experiments were performed in conformity with the Directive (2010/63/EU) of the European Parliament and of the Council and were approved by the Austrian Federal Ministry for Science, Research and Economy (BMWFW-66.019/0024-WF/V/3b/2016 and BMBWF-66.019/0036-V/3b/2018).

#### Groups for Behavioral and Histological Analyses

Female F344-rats of 10–12 weeks of age (140–190 g body weight) were purchased from Charles River Laboratories (Sulzfeld, Germany) and kept for at least 4 weeks in the animal facility to acclimatize to the handling by the experimenters. Prior to surgery, rats were randomly divided into three treatment groups that received, acutely after contusion and again 24 h later, either (a) 100 μL of Ringer-lactate (vehicle solution, *n* = 8), (b) 100 μL Ringer-lactate solution containing 10^6^ hUC-MSCs (*n* = 9), or (c) 100 μL of Ringer-lactate containing the extracellular vesicles secreted by 10^6^ hUC-MSCs within approximately 24 h (*n* = 9) via tail vein injections. Additionally, a fourth group (*n* = 8) was composed of sham-operated rats, which only received a laminectomy. Experimenters were blinded in regards to the content of injections and treatment groups until the end of the data acquisition and analysis.

#### Groups for mRNA Analysis

Rats were randomly divided into three treatment groups receiving acutely after contusion either (a) 100 μL of Ringer-lactate (vehicle solution, *n* = 6) or (b) 100 μL of Ringer-lactate containing the hUC-MSC-EVs secreted by 10^6^ hUC-MSCs (*n* = 6) via tail vein injections. Additionally, a third group (*n* = 6) was composed of sham-operated rats, only receiving a laminectomy. One day after injury or laminectomy, rats were deeply anesthetized by intraperitoneal injection of ketamine (273 mg/kg bodyweight), xylazine (7.1 mg/kg bodyweight), and acepromazine (0.625 mg/kg bodyweight), decapitated, and their spinal cords were dissected for mRNA extraction (see below).

### Surgeries

Analgesia was provided by subcutaneous (s.c.) injection of buprenorphine 0.03 mg/kg bodyweight 45 min prior to induction of operative narcosis with 1.8–2.5% isoflurane/O_2_. Body temperature was maintained at 37°C via a rectal probe-coupled heating pad and O_2_ saturation and pulse were monitored using a pulse-oxymeter (Emka Technologies). A dorsal laminectomy was performed at thoracic level 8 (Th8) leaving the exposed underlying dura mater intact. The neighboring vertebrae (Th7 and Th9) were fixed on the foramina intervertebralia using two Adson forceps. Using an impactor (Infinite Horizon, Precision System, and Instrumentation PSI), a contusion of 200 kdyn was applied on the exposed spinal cord at Th8 level and pressure and displacement of tissue were monitored. The rats belonging to the sham group underwent only a laminectomy. Post-operative analgesia was provided directly after surgery and daily for 5 days with meloxicam (1 mg/kg bodyweight s.c.). On the first 2 days post-surgery, rats additionally received buprenorphine (0.03 mg/kg bodyweight s.c.) twice per day. To prevent the occurrence of infection, enrofloxacin (10 mg/kg bodyweight) was administered s.c. on the day of surgery and daily until the 5th day post-OP. The bladder was manually voided 2–3 times per day. Rats with tSCI were housed on special soft bedding (Arbocell Comfort White bedding, Rettenmaier Austria GmbH). Food and water were freely accessible at a lowered height in the cages.

### Distribution of Intravenously Injected hUC-MSCs

The distribution of hUC-MSCs, and their possible accumulation at the lesion site, was assessed following intravenous application of 1 × 10^6^ hUC-MSCs fluorescently labeled with QTracker 625 (Thermo Fischer Scientific) in rats with either sham surgery or rats that received a tSCI 24 h before. One hour or 24 h after tail vein injection of labeled hUC-MSCs, the bulk of circulating cells was first removed by transcardial perfusion with 0.9% NaCl. Afterwards, rats were frozen in OCT embedding compound (Tissue-Tek, Sakura) for histological analysis. Whole body cross-sections were performed every 40 μm along the complete body axis, excluding the tail. The presence of labeled hUC-MSC was automatically detected and localized by microscopy (BioInvision Inc., Mayfield Village, OH, USA) (Supplementary Figure 1).

### Histology

On day 14 after surgery, rats were deeply anesthetized by intraperitoneal injection of ketamine (273 mg/kg bodyweight), xylazine (7.1 mg/kg bodyweight), and acepromazine (0.625 mg/kg bodyweight) and transcardially perfused with 0.9 % NaCl followed by 0.1 M phosphate-buffered 4% paraformaldehyde, pH 7.4. Following perfusion, spinal cords were prepared and further post-fixed for 1 h in 0.1 M phosphate-buffered 4% paraformaldehyde of pH 7.4 at room temperature. Tissues were then washed three times in PBS. A segment of 15 mm centered on the lesion was selected and transferred into 0.1 M phosphate-buffered 30% sucrose solution for 72 h. Then, samples were frozen in liquid nitrogen and embedded in OCT embedding compound (Tissue-Tek, Sakura). Using a cryostat (Leica CM1950), coronal sections of 15 μm of the spinal cord segments were collected in 10 series (each containing every 10th sections) on Superfrost Plus microscope slides (Thermo Scientific).

For immunohistological analyses, sections were washed with PBS + 0.1% Tween-20 (Sigma-Aldrich). The blocking solution was composed of PBS solution containing 1% bovine serum albumin fraction (Sigma-Aldrich), 0.2% fish skin gelatin (Sigma-Aldrich), and 0.1% Tween-20. The primary antibodies were guinea-pig anti-GFAP (1:500; Progen), goat anti-Iba1 (1:300; Abcam), rabbit anti-Collagen I (1:100; Abcam), goat anti-ChAT (1:100; Novus Biologicals), rabbit anti-NG2 chondroitin sulfate proteoglycan (1:200; Merck/Millipore), and rabbit anti-CD3 (1:150; Abcam). These were diluted in blocking solution and applied overnight at 4°C. The secondary antibodies were Alexa Fluor 568 donkey anti-rabbit (1:1,000; Invitrogen), Alexa Fluor 647 donkey anti-guinea pig (1:1,000; Dianova), and Alexa Fluor 568 donkey anti-goat (1:1,000, Molecular Probes), and these were applied overnight at 4°C. Nuclei were stained using 4′6-diamidino-2-phenylindole (DAPI; 0.5 μg/mL, Sigma-Aldrich). Finally, sections were mounted with fluorescent mounting medium (Dako) and were examined using an inverted fluorescence microscope (Olympus IX81) and a slide scanner (Olympus VS120).

### Histological Analyses

#### Spared Tissue

The volume of spared tissue was calculated for the region ranging from 3,150 μm caudal to 3,150 μm rostral from the epicenter. One section at every 1,050 μm of a series from the spinal cord tissues was processed for GFAP staining. Based on the detection of GFAP signal, the area of intact neural tissue was delineated on the micrographs using the Fiji ImageJ software (25). The total spared volume corresponded to the sum of the volumes extrapolated from the intact areas measured on each section and the distance between the sections (1,050 μm).

#### Cell Density

The density of Iba1^+^ cells was determined in two regions of interest—the gray matter of the ventral horn and the white matter of the anterior commissure—by counting all cells with a DAPI-labeled nucleus in a volume of 433 × 330 × 15 μm (section thickness) expressing Iba1. Since the intensity of the microglial response to the injury progressively decreased with distance from the epicenter, the densities were calculated on the sections located at 2 mm (rostral and caudal, one section each per rat) and at 3 mm (rostral and caudal, one section each per rat). Densities measured at the same distance on both sides of the lesion were pooled together. The tissue destruction at positions closer to the epicenter did not allow for reliable quantification in the two regions of interest. Additionally, corresponding positions of sham-group rats were analyzed along the rostral–caudal axis of the spinal cord.

The density of GFAP^+^ cells was evaluated in the ventral horn by counting all cells with a DAPI-labeled nucleus in a volume of 433 × 330 × 15 μm (section thickness) expressing GFAP. In the case of the GFAP^+^ cells, the response to injury was not significantly different at 2 and 3 mm, and therefore the densities measured at 2 and 3 mm were pooled together (four sections in total per rat). Due to their low abundance, the analysis of CD3^+^ cells density was conducted within the whole area of gray matter on each section analyzed. Sections located at 2 and 3 mm, both rostral and caudal from the epicenter, were measured and pooled together (four sections in total per rat). Additionally, corresponding positions of sham-group rats were analyzed along the rostral–caudal axis of the spinal cord.

#### Expression Level

GFAP, NG2- chondroitin sulfate proteoglycan and collagen I expressions were quantified according to the area stained. Immunofluorescence images were acquired with fixed parameters and binarized. The area covered by the staining was determined using the Fiji ImageJ software (25). For the quantification of GFAP expression, measurements of one ventral horn were performed on sections located at 2 and 3 mm, both rostral and caudal, from the injury epicenter and pooled together (four sections in total per rat). The ventral horn could not be reliably defined in regions closer to the epicenter. For the quantification of collagen I and NG2- chondroitin sulfate proteoglycan expression, the total area stained on one section located at every 1,050 μm from 3,150 μm caudal to 3,150 μm rostral to the epicenter was quantified. Additionally, corresponding positions of sham-group rats were analyzed along the rostral–caudal axis of the spinal cord.

#### Particle Size Analysis

The distribution of size of Iba1-expressing cells was also evaluated in the gray matter of the ventral horn and the white matter of the anterior commissure from the micrographs used to calculate Iba1 cell density (see above). The analyses were conducted on sections located 2 and 3 mm, both rostral and caudal, from the injury epicenter of each rat, and the results were pooled together. The cell size, precisely the area covered by Iba1 staining, was determined with the Fiji ImageJ software (25), applying a constant threshold for brightness and contrast as well as size and circularity. Additionally, corresponding positions of sham group rats were analyzed along the rostral–caudal axis of the spinal cord.

#### Number of ChAT^+^ Cells

The number of ChAT^+^ cells was determined on sections located 2 and 3 mm, both rostral and caudal, from the injury epicenter of each rat. The number of ChAT^+^ cells was counted in the two ventral horns of the spinal cord, and the results were pooled together (four sections per rat). Additionally, corresponding positions of sham-group rats were analyzed along the rostral–caudal axis of the spinal cord.

### Primary Microglia Culture and mRNA Expression Analysis

Mixed glia cultures were produced as described (26). Mixed glia cultures were obtained from brains of Fischer-344 rats at post-natal day 1. Post-natal pups were decapitated, and the brains were removed and collected in RPMI medium (ThermoFischer). After carefully removing the meninges, the brains were transferred into a dish containing “mixed glia medium”: RPMI medium (ThermoFisher), 10% fetal bovine serum (FisherScientific), 2 mM L-Glutamine (FisherScientific), and 100 U/mL penicillin and 100 μg/mL streptomycin (HVD), dissociated by gentle trituration with a 10 mL pipette, and seeded in T75 cell culture flasks (one brain per flask). Prior to seeding, the flasks were coated with 5 μg/mL poly-D-lysine (Sigma-Aldrich) in PBS. The cultures were maintained in mixed glia medium at 37°C and 5% CO_2_. The medium was replaced 1 day after seeding and then every other day. After 7 days of culture, the mixed glia cells formed a confluent layer consisting of astrocytes, some ependymal cells, O2A precursor cells, and microglia. To detach the loosely adherent microglia from the confluent cell layer, the culture flasks were shaken at 220 rpm for at least 2 h at 37°C. Afterwards, the supernatant was collected and microglia were given 1 h to adhere on culture dishes at 37°C and 5% CO_2_ before medium was replaced and non-adherent cells removed. The adherent microglia were carefully detached using mixed glia medium and seeded in six-well plates at 5 × 10^5^ cells/well. The purity of the microglial-enriched culture (>95%) was confirmed by the detection of Iba1 by immunocytochemistry and CD11b/CD45 by flow cytometry measurements (data not shown).

To activate microglia, 10 ng/mL lipopolysaccharide (LPS, Sigma-Aldrich) was added to the medium for 24 h. Alternatively, PBS was added as vehicle control. The impact of hUC-MSC-EVs on microglial activation was scrutinized by applying EVs secreted by 4 × 10^5^ hUC-MSCs in each well-immediately after LPS. PBS served as a control.

Total RNA was prepared from microglia using the TRIzol reagent (Sigma-Aldrich) according to the manufacturer's protocol. Total RNA concentrations were determined with a NanoVue plus (GE Healthcare, UK). RNA was reverse transcribed into first-strand cDNA using the iScript TM Reverse Transcription Supermix for RT-qPCR (Bio-Rad Laboratories, CA) according to the manufacturer's protocol. Quantitative gene expression analyses were performed using TaqMan RT-PCR technology. Technical duplicates containing 10 ng of reverse-transcribed RNA were amplified with the GoTaq Probe qPCR Master Mix (Promega) using a two-step cycling protocol (95°C for 15 s, 60°C for 60 s; 50 cycles, Bio-Rad CFX 96 Cycler). Several validated exon-spanning gene expression assays were employed: pPIA Rn.PT.39a.22214830 and TBP Rn.PT.39a.22214837 from Integrated DNA Technologies (IDT); IL-6 Rn01410330 and NLRP3 Rn04244620 from ThermoFisher; and IL-1β NM_031512.2 from Sino Biological.

The relative expression levels of the target genes were normalized on two validated housekeeping genes, pPIA and TBP. Cq values were analyzed using qBasePlus v. 3.2 (Biogazelle NV, Zwijnaarde, Belgium). The expression of target genes in control and treatment conditions was normalized to represent the relative expression in terms of “fold changes.”

### mRNA Expression Analysis at the Lesion Site After Spinal Cord Injury

Total RNA of each spinal cord was isolated from a 1 cm segment centered on the lesion site, or the corresponding position in the sham group, using TRIzol reagent (Sigma-Aldrich) according to the manufacturer's protocol. Total RNA concentrations were determined with a NanoVue plus (GE Healthcare, UK). RNA was transcribed into first-strand cDNA using the iScript TM Reverse Transcription Supermix for RT-qPCR (Bio-Rad Laboratories, CA) according to the manufacturer's protocol. Quantitative gene expression analyses were performed using TaqMan RT-PCR technology. Technical duplicates containing 10 ng of transcribed RNA were amplified with the GoTaq Probe qPCR Master Mix (Promega) using a two-step cycling protocol (95°C for 15 s, 60°C for 60 s; 50 cycles, Bio-Rad CFX 96 Cycler). Several validated exon-spanning gene expression assays were employed: pPIA Rn.PT.39a.22214830 and TBP Rn.PT.39a.22214837 from Integrated DNA Technologies (IDT); IL-6 Rn01410330 and NLRP3 Rn04244620 from ThermoFisher; and IL-1β NM_031512.2 from Sino Biological.

The relative expression levels of the target genes were normalized on two validated housekeeping genes, pPIA and TBP. Cq values were analyzed using qBasePlus v. 3.2 (Biogazelle NV, Zwijnaarde, Belgium). The expression of target genes in control and treatment conditions was normalized to represent the relative expression in terms of “fold changes.”

### Statistics

Statistical analyses were performed using the Prism 8 software (GraphPad). The validation of the normal distribution of data allowed for the use of parametric tests. One-way ANOVA tests were performed to compare the results between the various treatment groups, and this was followed by a Bonferroni *post-hoc* test. Repeated measurement one-way ANOVA was used for the analysis of gene expression in rat primary microglia *in vitro*; this analysis was followed by Tukey *post-hoc* test. Statistical significance was assumed for *p* ≤ 0.05.

## Results

### Early Application of hUC-MSCs and hUC-MSC-EVs Quenches the Inflammatory Response After tSCI

We compared the impact of early intravenous delivery of hUC-MSCs, with the one of hUC-MSC-EVs, on secondary damages in a rat model of tSCI. Rats received a contusion at the thoracic level 8 (Th8), generating a middle to severe spinal cord injury characterized by significant loss of parenchyma at the epicenter of the lesion and the maintenance of a thin rim of white matter. Either 1 × 10^6^ hUC-MSCs, the recovered EVs secreted by 1 × 10^6^ hUC-MSCs, or the vehicle solution were injected via the tail vein of the rats. Intravenous application was conducted immediately after tSCI and was repeated 24 h post-injury.

**Figure 1:**
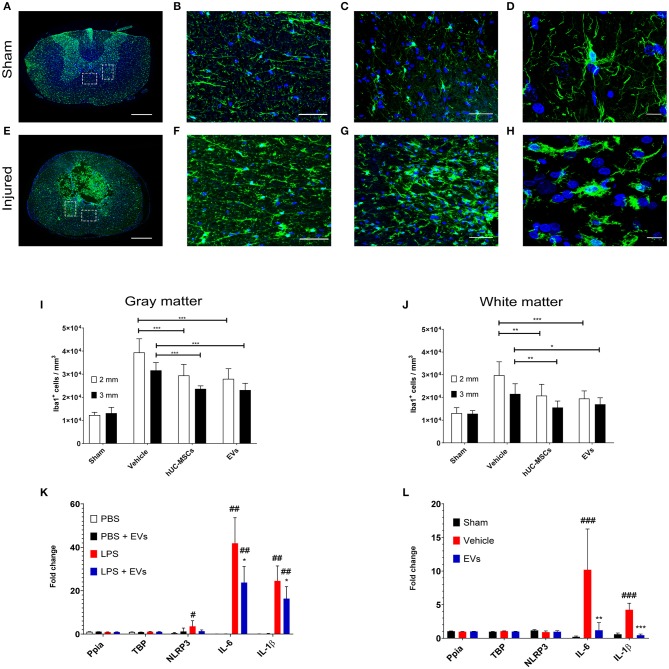
Representative micrographs of the spinal cord of rats from the sham group **(A–D)** and rats which received a spinal cord injury (2 mm rostral from the epicenter) **(E–H)**. Scale bar in **(A,E)** = 500 μm. Scale bar in **(B,C,F,G)** = 50 μm. Scale bar in **(D,H)** = 10 μm. **(A,E)** Immunodetection of Iba1-expressing microglia (green). **(B,F)** larger magnifications of the dashed lined area of the anterior commissure shown in **(A,E)**. **(C,G)** larger magnifications of the dashed lined area of the ventral horn shown in **(A,E)**. **(D)** The surveillance microglia shows a small soma size with long filigreed processes. **(H)** Activated microglia exhibits an amoeboid morphology with a larger soma and few stunted processes. **(A–H)** Nuclear counterstain was obtained with DAPI (blue). The density of Iba1-expressing cells was quantified 2 weeks after tSCI at 2 mm and 3 mm both rostral and caudal from the lesion epicenter in **(I)** the ventral horn and in **(J)** the anterior commissure. The equivalent positions along the rostro–caudal axis were used to measure the density of Iba1-expressing cells in the spinal cord of the sham group. **(I,J)** Groups were compared using one-way ANOVA and Bonferroni *post-hoc* test. Statistical significances of column linked by horizontal lines *p ≤ 0.05; **p ≤ 0.01; and ***p ≤ 0.001. **(K)** Impact of hUC-MSC-EVs on the expression level of pro-inflammatory genes after LPS activation in rat primary microglia. Ppia and TBP were used as non-regulated genes for standardization. Groups were compared using repeated measurement one-way ANOVA and Tukey *post-hoc* test. Statistical significances compared to the PBS group ^#^*p* ≤ 0.05; ^##^*p* ≤ 0.01; statistical significances compared to the LPS group *p ≤ 0.05. **(L)** Impact of hUC-MSC-EVs on the expression level of pro-inflammatory genes after tSCI. Ppia and TBP were used as non-regulated genes for standardization. Groups were compared using one-way ANOVA and Bonferroni *post-hoc* test. Statistical significances compared to the sham group ^###^*p* ≤ 0.001; statistical significances compared to the vehicle group **p ≤ 0.01; ***p ≤ 0.001.

Fourteen days following contusion, a significant increase in the density of Iba1-expressing microglia/macrophages was detected in the lesioned spinal cord, as compared to the situation observed in sham-operated rats (one-way ANOVA, *p* < 0.001) (Figures 1A–J). The accumulation of Iba1^+^ cells was highest at the lesion epicenter and progressively diminished with increasing distance from the lesion epicenter. Due to the importance and the variability of tissue destruction close to the epicenter, the density of Iba1-expressing cells was quantified at 2 and 3 mm rostral and caudal from the injury epicenter. As shown in Figures 1I,J (Table 1), an early intravenous application of hUC-MSCs or hUC-MSC-EVs significantly dampened the accumulation of Iba1^+^ cells by ~25–30% in both gray and white matter following tSCI, as compared to vehicle treatment. Intravenous application of either hUC-MSCs or hUC-MSC-EV was equally effective to counteract the massive accumulation of Iba1^+^ cells. The densities of Iba1^+^ cells in the three groups bearing a tSCI were significantly higher than in the sham group (one-way ANOVA, *p* < 0.001). The density of Iba1-expressing microglia in the sham group was constant along the spinal cord.

**Table 1:** Summary of histological measurements for each group.

	**Sham**	**Vehicle**	**hUC-MSCs**	**hUC-MSC-EVs**
**Iba1**^+^ **cells/mm**^3^				
Gray matter				
2 mm	12,276 ± 1,234	39,410 ± 5,922^###^	29,419 ± 4,819***, ^###^	27,955 ± 4,393***, ^###^
3 mm	13,112 ± 2,521	31,653 ± 3,327^###^	23,652 ± 1,285***, ^###^	23,108 ± 2,957 ***, ^###^
White matter				
2 mm	13,078 ± 2,399	29,772 ± 5,941^###^	20,788 ± 5,005**, ^###^	19,518 ± 3,372***, ^###^
3 mm	12,830 ± 1,386	21,549 ± 4,476^###^	15,578 ± 2,850**, ^###^	16,964 ± 2,892*, ^###^
**Freq. Iba1**^+^ **cells**				
Gray matter				
<150 μm^2^	0.56 ± 0.04	0.75 ± 0.04^###^	0.76 ± 0.05^n.s., ###^	0.75 ± 0.04^n.s., ###^
≥150 μm^2^	0.43 ± 0.04	0.25 ± 0.04^###^	0.24 ± 0.05^n.s., ###^	0.25 ± 0.04^n.s., ###^
White matter				
<150 μm^2^	0.56 ± 0.04	0.75 ± 0.03^###^	0.76 ± 0.04^n.s., ###^	0.75 ± 0.04^n.s., ###^
≥150 μm^2^	0.43 ± 0.04	0.25 ± 0.04^###^	0.24 ± 0.04^n.s., ###^	0.24 ± 0.04^n.s., ###^
**CD3**^+^**cells/mm**^3^	46.8 ± 7.4	294.8 ± 82.1^###^	285.9 ± 62.6^n.s., ###^	220.5 ± 57.2^n.s., ###^
**GFAP**^+^ **cells/mm**^3^	17,178 ± 2,243	19,908 ± 5,212^n.s.^	20,228 ± 5,613^n.s., n.s.^	20,541 ± 3,521^n.s., n.s.^
**% GFAP**^+^ **Area**	2.2 ± 0.7	14.4 ± 8.6^###^	5.5 ± 3.2^**, *n*.*s*.^	5.7 ± 2.5^**, *n*.*s*.^
**Total Collagen I area (**μ**m**^2^**)**	29,011 ± 15,903	2,203,763 ± 936,900^###^	1,503,775 ± 853,435^n.s., ##^	959,715 ± 560,646 ^**, *n*.*s*.^
**Spared tissue volume (mm**^3^**)**	38.3 ± 3.05	21.2 ± 2.2^###^	20.8 ± 3.7^n.s., ###^	21.6 ± 3.0^n.s., ###^
**Number ChAT**^+^ **cells**	52.3 ± 5.8	32.4 ± 6.4^#, n.s.^	36.4 ± 11.1^n.s., n.s.^	38.2 ± 9.8^n.s., n.s.^
**Total CSPG-NG2 area (**μ**m**^2^**)**	538,731 ± 43,789	3,690,575 ± 527,086^###^	2,993,413 ± 637,420^###, n.s.^	2,735,803 ± 682,481^###, *^

Statistical significant differences to the sham group are marked with

# p ≤ 0.05;

## p ≤ 0.01; and

### p ≤ 0.001. Statistical significant to differences to the vehicle are marked with

* p ≤ 0.05;

** p ≤ 0.01; and

*** *p ≤ 0.001. Non-significant differences are marked with n.s*.

A change in cell morphology from large ramified to small amoeboid (Figures 1D,H) is a hallmark of microglial activation in response to injury (27). Accordingly, the size of Iba1-expressing cells was analyzed to address the impact of hUC-MSCs and hUC-MSC-EVs on microglial activation after contusion. A higher frequency of small Iba1^+^ cells (i.e., <150 μm^2^) was observed after tSCI as compared to the sham condition (Supplementary Figures 2A,B; Table 1). However, based on the distribution of their size, no differences were recognized at 14 days post-injury between the three lesioned groups (Supplementary Figures 2A,B). Thus, the early intravenous application of hUC-MSCs or hUC-MSC-EV after tSCI significantly decreased the accumulation of Iba1-expressing cells but not their activation state in response to injury.

Following tSCI, the rapid upregulation of IL-1α and IL-1β secretion by activated microglia triggers the infiltration of leukocytes and sustains inflammatory processes (4, 28). Three days post-injury, the number of recruited neutrophils and T-lymphocytes at the lesion site reaches a maximum (7). Although neutrophils rapidly disappear from the lesion site thereafter, residual T-lymphocytes persist for several weeks. Accordingly, no neutrophils were detected in the spinal cord 14 days after tSCI, irrespective of the treatment administered (data not shown). In contrast, CD3-expressing T-lymphocytes were significantly more abundant in the lesioned parenchyma when compared to the intact parenchyma of the sham group (Supplementary Figure 2C; Table 1). The early application of hUC-MSCs or hUC-MSC-EVs after tSCI had no impact on the number of T-lymphocytes detected as compared to rats that received only the vehicle.

### hUC-MSC-EVs Can Directly Interact With Activated Rat Primary Microglia

As demonstrated by the distribution of intravenously injected hUC-MSCs (Supplementary Figure 1), the modulation of inflammation obtained after the injection of hUC-MSCs was unlikely to be the result of direct interaction with the lesioned tissue, but, rather, it originated from secreted factors, such as EVs. EVs secreted by the injected hUC-MSCs or the injected hUC-MSC-EVs may have the potential to reach the lesioned spinal tissue and directly interact with microglia. The capacity of hUC-MSC-EVs to interact with microglia was addressed *in vitro* using rat primary microglia cultures. The activation of rat primary microglia with lipopolysaccharides (LPS) rapidly upregulates gene expression of the pro-inflammatory cytokines IL-1β, IL-6, and the inflammasome component NLRP3 (Figure 1K). The application of hUC-MSC-EVs significantly reduced the induction of IL-1β and IL-6 expression in rat primary microglia in response to LPS (Figure 1K). Thus, EVs derived from hUC-MSCs may directly interact with microglia during their early activation response and modify the profile of cytokine expression toward a milder pro-inflammatory status.

The observation that hUC-MSC-EVs decreased the expression of pro-inflammatory cytokines in activated microglia *in vitro* was further validated *in vivo* following tSCI. Previous reports showed that the induction of IL-1β and IL-6 expression is a very early event following tSCI [see, for example, Pineau and Lacroix (28)]. We therefore addressed the impact of acute hUC-MSC-EVs' intravenous application on the expression of these pro-inflammatory cytokines at 24 h post-lesion (Figure 1L) and confirmed that hUC-MSC-EVs can also significantly reduce the expression of IL-1β and IL-6 within the lesioned spinal cord and thereby contribute to a milder pro-inflammatory environment.

### Reduced Scarring Response to tSCI Following Early Application of hUC-MSCs or hUC-MSC-EVs

**Figure 2:**
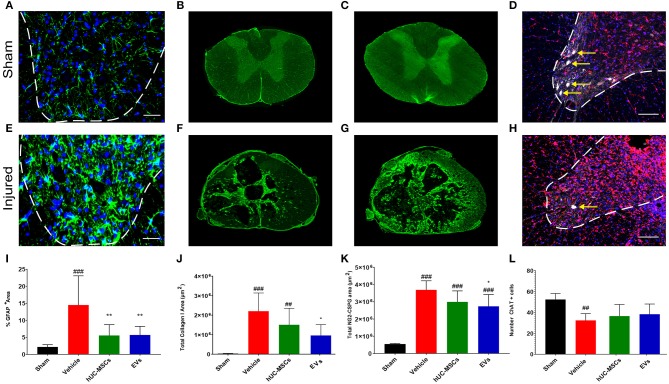
Immunodetection of various markers expressed in the spinal cord of a sham rat **(A–D)** and in a rat following tSCI **(E–H)**. Immunodetection of GFAP-expressing astrocytes in the ventral horn of sham operated and injured rats (2 mm rostral from the epicenter) **(A,E)**. Scale bar **(A,E)** = 50 μm, ventral horn deliminated with dashed lines. Immunodetection of Collagen I in the spinal cord of sham and injured rats (lesion epicenter) **(B,F)**. Immunodetection of NG2-CSPGs in the spinal cord of sham and injured rats (lesion epicenter) **(C,G)**. Immunodetection of ChAT-expressing motor neurons (white, yellow arrows) and Iba1-expressing microglia (red) in the spinal cord of sham and injured rats (2 mm rostral from the epicenter) **(D,H)**. Nuclear counterstain with DAPI (blue). Scale bar **(D,H)** = 100 μm, ventral horn deliminated with dashed lines. **(I)** Percentage of area covered by GFAP at 2 and 3 mm rostral and caudal from the lesion epicenter in the ventral horn. Total area covered by Collagen I **(J)** and NG2-CSPG **(K)** depositions were quantified on spinal cord sections ranging from 3 mm rostral to 3 mm caudal from the lesion epicenter. The equivalent positions along the rostro–caudal axis were used to measure the area covered by collagen I and CSPG-NG2 in the spinal cord of the sham group. **(L)** Total number of ChAT^+^ motor neurons quantified in the ventral horns of the spinal cords at 2 and 3 mm both rostral and caudal from the lesion epicenter and at the equivalent positions along the rostro–caudal axis of the sham operated rats. **(I–L)** Groups were compared using one-way ANOVA and Bonferroni *post-hoc* test. Statistical significances compared to sham group: ^##^*p* ≤ 0.01; ^###^*p* ≤ 0.001, statistical significances compared to vehicle group: *p ≤ 0.05; and **p ≤ 0.01.

During the sub-acute phase post-tSCI [from ~2 days to 2 weeks after injury, (2)], the activation and proliferation of astrocytes around the lesion site contributes to the development of the fibroglial scar. Reactive astrogliosis after tSCI is an essential protective mechanism to minimize and seal the lesion. However, following the initial response to injury, reactive astrocytes secrete various inhibitory components that impede or even block regenerative processes (29). Reactive astrocytes can be distinguished through their upregulation of glial fibrillary acid protein (GFAP) expression, giving them an hypertrophic appearance (Figures 2A,E) (29, 30). Fourteen days post-tSCI, no increase in the density of GFAP^+^ cells was detected in the gray matter of lesioned spinal cords, as compared to intact tissue of sham rats (Supplementary Figure 2D). However, after tSCI, the morphological changes associated with reactive gliosis led to a strong augmentation of the area covered by GFAP (Figures 2A,E,I; Table 1). The early intravenous application of hUC-MSCs or hUC-MSC-EVs significantly lowered the expression of GFAP after tSCI, as compared to the vehicle treatment (Figure 2I). Moreover, only rats that received the vehicle treatment had significantly increased GFAP-labeling as compared to the sham group.

While astrocytes are major contributors of fibroglial scar formation, the deposition of non-cellular components in the extracellular space, such as collagen and CSPGs, is pivotal (31). Whereas, astrocytes are inherent supportive glial cells of the neural tissue, collagen I accumulation is only observed following injury of the CNS. To assess the impact of early intravenous injection of either hUC-MSCs or hUC-MSC-EVs on the extent of scarring, the accumulation of collagen I in the injured spinal cord was histologically analyzed. Collagen I immunoreactivity was barely detectable in the spinal cord of rats belonging to the sham group (Figures 2B,J). By contrast, noticeable accumulation of collagen I was detected in rats that received only the vehicle treatment following tSCI (Figure 2F). Following intravenous application of hUC-MSCs or hUC-MSC-EVs, the extent of pathological accumulation of collagen I in response to tSCI was reduced by 32 and 56%, respectively (Figure 2J). Similarly, tSCI provoked the accumulation of NG2-CSPGs at the lesion site (Figures 2C,G). Again, the intravenous application of hUC-MSCs or hUC-MSC-EVs significantly reduced the deposition of NG2-CSPGs in the extracellular matrix by 19 and 26%, respectively, as compared to rats treated with the vehicle (Figure 2K). Hence, 14 days post-injury, astrogliosis and scarring were significantly reduced by the early intravenous application of hUC-MSC-EVs and to a lesser extent by hUC-MSCs, as compared to vehicle treatment.

### Short Term Outcomes Following Early Application of hUC-MSCs or EVs After tSCI

Primary and secondary damages ensuing from tSCI cause the destruction of spinal tissue and the formation of cavities or cysts (1, 2). Although the trauma that causes the primary damage in tSCI is unforeseeable and irreversible, secondary damages are caused by processes that persist for a few weeks in tSCI rodent models and likely up to a year or more in humans (1, 2). The period of secondary damages presents a time window in which therapeutic interventions can be applied to stop or reduce secondary degradation of the parenchyma.

Based on the detection of choline acetyltransferase (ChAT) in motor neurons of the ventral horn, we assessed whether the application of hUC-MSCs or hUC-MSC-EVs acutely after tSCI would have improved their survival. As shown in Figures 2D,H,L (Table 1), tSCI caused in rats receiving the vehicle a significant decrease in the number of motor neurons that could be detected in the ventral horn in the vicinity of the lesion site (2–3 mm) at 14 days post-injury. Following intravenous application of hUC-MSCs or hUC-MSC-EVs, a slightly higher number of ChAT^+^ motor neurons were detected; however, the differences were neither significant compared to the sham group nor to the vehicle group (Figure 2L; Table 1).

**Figure 3:**
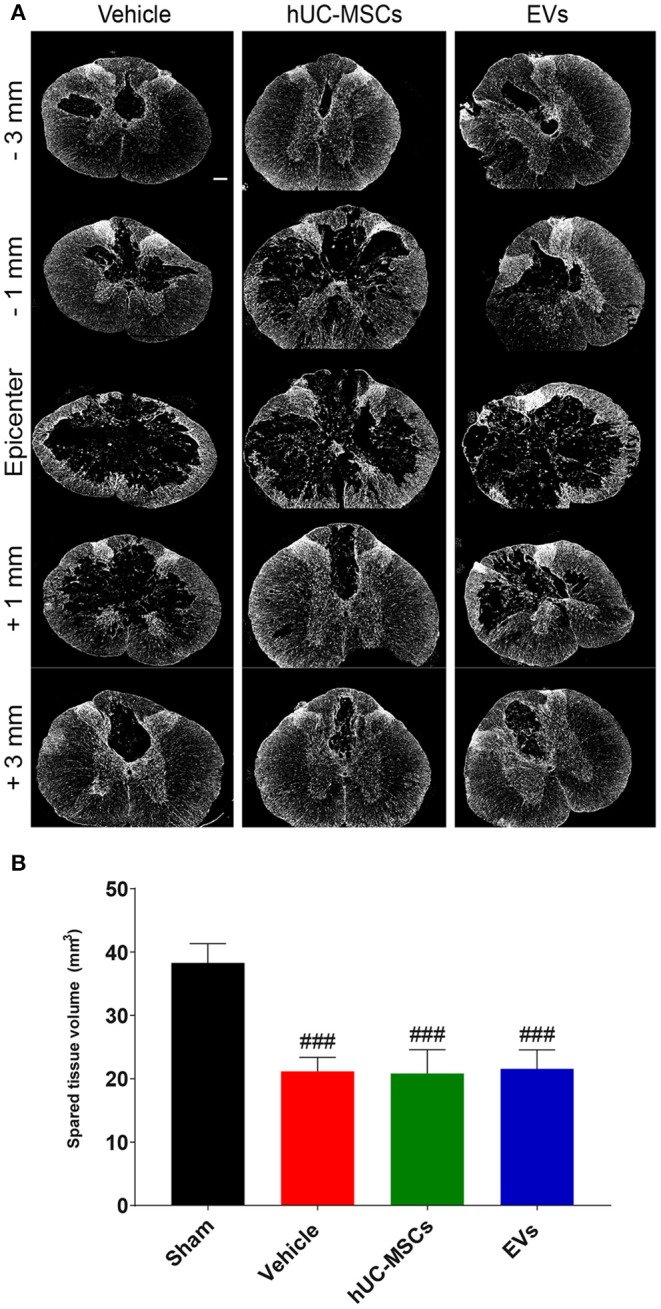
**(A)** Representative immunodetection of GFAP on cross-sections along the rostro–caudal axis of the spinal cord, 2 weeks after tSCI. Scale bar = 200 μm. **(B)** Total volume of spared spinal cord parenchyma calculated from 3 mm rostral to 3 mm caudal to the lesion epicenter or the equivalent position in the sham rats. Groups were compared using one-way ANOVA and Bonferroni *post-hoc* test. Statistical significances compared to sham group: ^###^*p* ≤ 0.001.

Using the GFAP-immunoreactivity as a tool to distinguish intact neural tissue from necrotic or scarred tissue and cysts, the volume of the remaining intact spinal cord was assessed 14 days post-injury from 3 mm rostral to 3 mm caudal of the lesion epicenter (Figures 3A,B; Table 1). Rats treated with the vehicle lost approximately 45% of their spinal parenchyma in the segment of ~6 mm centered on the lesion, as compared to the sham group. A comparison revealed no significant difference in the volume of spared parenchyma between the rats injected with hUC-MSCs, hUC-MSC-EVs, or vehicle.

## Discussion

The induction of the inflammatory processes after tSCI is a very early and pivotal initiating event for the course of secondary damages. Therefore, anti-inflammatory interventions are required as swiftly as possible. The early inflammatory response is triggered within minutes to hours after lesion and involves primarily microglia, neutrophils, and macrophages (7, 28, 32, 33). Within the first days of injury, activated immune cells are responsible for the clearing of cellular debris at the lesion site (34). Thus, a complete abolition of early microglial activation after tSCI would be detrimental to the course of recovery. Unfortunately, inflammation does not resolve and becomes a deleterious chronic process hindering recovery and regeneration (7). Previous studies involving animal models and humans have proposed that the effectiveness of MSCs to control inflammatory processes is driven by paracrine signaling on immune cells (35). Here, we have demonstrated in a tSCI model that EVs secreted by hUC-MSCs carry anti-inflammatory and anti-scarring activities resembling the ones of their parental MSCs.

The swift intravenous application of EVs following tSCI allows for the distribution of a pleiotropic therapeutic agent that targets multiple sites simultaneously. We confirmed *in vitro* that hUC-MSC-EVs can interact with activated rat primary microglia directly and quench the expression of pro-inflammatory cytokines characterizing the early phase of secondary damages. The expression of the initiating cytokines IL-1β and IL-6 exhibited the strongest decrease upon hUC-MSC-EVs application. Recent studies also reported a significant decrease of pro-inflammatory cytokine expression and secretion in BV-2 cells as well as primary macrophages and microglial cells following the application of MSC-EVs (36–38). Furthermore, we demonstrated the capacity of hUC-MSC-EVs to reduce *in vivo* as well the expression of cytokines IL-1β and IL-6 in the spinal cord at 24 h post-injury, which resulted in a milder inflammatory environment.

Based on morphological analyses, we found that the activation capacity of Iba1-expressing cells *in vivo* was not compromised by the systemic application of hUC-MSC-EVs or whole hUC-MSCs. Hence, whereas the expression of pro-inflammatory cytokines and the density of immune cells in the vicinity of the lesion were significantly decreased by the application of hUC-MSC-EVs and hUC-MSCs, the acute immune response to tSCI was not silenced. This observation complements a previous report that used flow cytometry to show that a single intravenous application of hMSC-EVs can diminish the number of microglia residing in the spinal cord at 14 days following tSCI (39). The roles of microglia and macrophages following injury to the CNS are extremely complex and can be detrimental as well as pro-regenerative (5). Lankford et al. recently observed that, following intravenous administration of labeled bone marrow-derived MSC-EVs in a rat model of tSCI, the EVs were detected almost exclusively in CD206-expressing macrophages, i.e., pro-regenerative macrophages (40). It remains to be clarified whether EVs are selectively uptaken by macrophages expressing CD206, or if the presence of internalized MSC-EVs rapidly convert the phenotype of macrophages toward a pro-regenerative one. The phenotype of activated Iba1-expressing cells following the application of hUC-MSC-EVs and hUC-MSCs, i.e., pro-inflammatory, pro-regenerative, or a mixture of both, remains to be determined.

Astrocytes, along with pericytes and fibroblasts, are major components involved in scar formation after SCI (29). Scarring is crucial to seal the lesion site following injury and to restore tissue integrity. However, intense scarring is considered to hinder axonal regeneration and functional connectivity of potential grafted relay neurons (29). In our tSCI model, the application of hUC-MSC-EVs or hUC-MSCs diminished the activation of reactive astrogliosis by roughly 40%. In parallel, application of hUC-MSC-EVs significantly reduced the deposition of collagen I and NG2 at the lesion site, which, together with reduced astrogliosis, indicated an anti-scarring activity. Acute application of hUC-MSCs, or their secreted hUC-MSC-EVs, did not significantly influence the number of surviving motor neurons nor the amount of spinal cord tissue spared at the lesion site 14 days following tSCI. Although further damage and tissue re-organization takes place for at least 8 weeks post-injury, we concluded from our observation that intravenous application of hUC-MSCs or hUC-MSC-EVs does not diminish the acute tissue damage associated with contusion.

The results presented here are indicative of the beneficial effect of MSC-EV application following tSCI. Recent studies reported improvement of long-term functional outcome using various models of spinal cord injury and types of EVs preparation (18, 37, 41, 42). These studies demonstrated that the positive impact of intravenous MSC-EVs applications in tSCI models is, at least in part, associated with their anti-inflammatory activities. Moreover, functional improvements were significant in the late sub-acute phase (>14 days), which suggested that intravenous MSC-EVs application provided a better environment for functional recovery.

Regarding the potential mode of action for MSC therapy, we have recently observed a remarkable paradigm shift that claims that cell-derived bioactive molecules support endogenous repair processes despite a lack of engraftment of transplanted cells (43). There is also an increase in published reports confirming that the effects of MSCs can be recapitulated by the EVs that are secreted during *in vitro* cell expansion [reviewed in (43–45)]. Although many details underlying the paracrine effect that are mediated by MSC-EVs remain to be uncovered, the impact of the paracrine hypothesis for the development of novel therapies for regenerative medicine, and specifically for traumatic spinal cord injury, is obvious.

In summary, we demonstrated that the anti-inflammatory and anti-scarring activity associated with an intravenous administration of MSCs in a model of tSCI can be obtained, and even surpassed, by the sole application of the EVs they secrete. Although the experimental paradigm employed in this study did not reveal neuroprotection, the environment of the lesion observed following hUC-MSCs or hUC-MSC-EVs application may be more suitable for long-term regenerative processes, or regenerative therapies to be applied in combination. Due to their significant advantages related to manufacturing, storage, and application, the acute administration of MSC-EVs constitutes a promising future avenue for the treatment of patients affected by tSCI.

## Data Availability Statement

All datasets generated for this study are included in the article/Supplementary Material.

## Ethics Statement

The animal study was reviewed and approved in conformity with the Directive (2010/63/EU) of the European Parliament and of the Council and were approved by the Austrian Federal Ministry for Science, Research and Economy (BMWFW-66.019/0024-WF/V/3b/2016 and BMBWF-66.019/0036-V/3b/2018).

## Author Contributions

PR, LB, CS, KP, CK, PZ, and DJ performed the experimental work. PR, LB, CS, KP, CK, PZ, DJ, HM, BB, FR, LA, ER, MG, DS, and SC-D designed the experiment and interpreted the results. PR, LA, ER, MG, DS, and SC-D wrote the manuscript.

### Conflict of Interest

The authors declare that the research was conducted in the absence of any commercial or financial relationships that could be construed as a potential conflict of interest.
