# Supplementary material for: Extracellular Vesicles Can Deliver Anti-inflammatory and Anti-scarring Activities of Mesenchymal Stromal Cells After Spinal Cord Injury
Source: Front Neurol. 2019 Nov 29;10:1225. doi: 10.3389/fneur.2019.01225 (PMC6896947; doi:10.3389/fneur.2019.01225)
Supplement: Supplementary file 1 [file Data_Sheet_1.docx]

**Supplementary Material**

**hUC-MSCs accumulate in filtrating organs shortly after intravenous injections**

Fluorescently labelled hUC-MSCs were injected intravenously in rats with tSCI and sham animals to determine whether hUC-MSCs accumulate in the spinal cord, especially following lesion. Rats received a contusion at the thoracic level 8 (Th8) and on the following day, 1 x 10^6^ labelled hUC-MSCs were injected intravenously. One hour or 24 hours after cell injection, whole body cross-sections were performed along the complete body axis to localise the labelled hUC-MSCs. In agreement with previous reports (de Witte et al., 2018; Saat et al., 2016), we observed a rapid clearance of the hUC-MSCs from the circulation. One hour after intravenous application, more than 90% of the labelled hUC-MSCs were detected in the lungs and the liver (Supp. Fig. 1). Only 15% of the hUC-MSCs were detected 24 hours after application and more than 75% of these cells were still localised in the lungs and the liver. hUC-MSCs were neither detected in the intact nor in the injured spinal cord parenchyma at the two time-points after injection.

**Supplementary Figure Legend**

**Suppl Fig. 1**

3D reconstructions of serial cross-sections of a sham rat one hour after intravenous injection of 1 x 10^6^ fluorescently labeled hUC-MSCs (A and C). 3D reconstruction of serial cross-sections of a rat with tSCI twenty-four hours after intravenous injection of 1 x 10^6^ fluorescently labeled hUC-MSCs (B and D). Detected hUC-MSC are depicted as yellow dots and the positions of the lung (red), liver (green) and spleen (blue) are schematically represented. Magnifications showing the accumulation of labeled hUC-MSCs (violet) in the lungs of rat one hour (E) and twenty-four hours (F) after intravenous injection. Magnifications showing the spinal cord (dashed line area) twenty-four hour after tSCI under bright-field illumination (G) and the absence of labeled hUC-MSCs (violet) in the spinal cord one hour after intravenous injection (H). A few labelled hUC-MSCs were detected in the neighboring tissues (arrows).

**Suppl Fig. 2**

Distribution of the size of Iba1-expressing cells in the ventral horn (A) and in the anterior commissure (B). C) Average density of CD3-expressing T-lymphocytes detected in the spinal cord 2 weeks after tSCI at 2 mm and 3 mm, rostral and caudal, from the lesion epicenter. D) Average density of GFAP-expressing cells was quantified 2 weeks after tSCI at 2 mm and 3 mm, rostral and caudal, from the lesion epicenter in the ventral horn. Groups were compared using one-way ANOVA and Bonferroni post-hoc test. Statistical significances compared to the sham group: (_###_) p ≤ 0.001.
