# Supplementary figures and images for: Extracellular Vesicles Can Deliver Anti-inflammatory and Anti-scarring Activities of Mesenchymal Stromal Cells After Spinal Cord Injury
Source: Front Neurol. 2019 Nov 29;10:1225. doi: 10.3389/fneur.2019.01225 (PMC6896947; doi:10.3389/fneur.2019.01225)

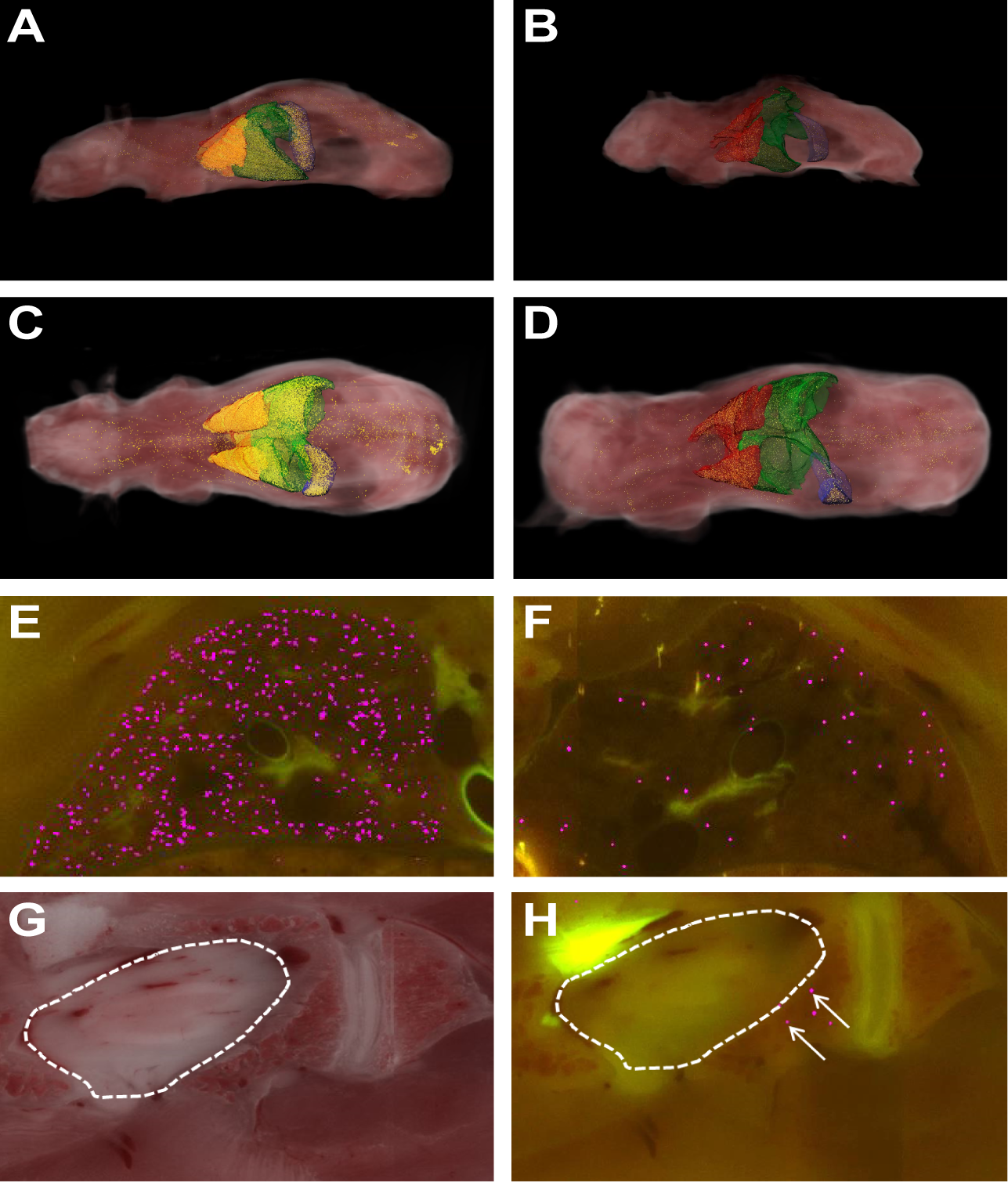

Supplement: Supplementary file 2 [file Image_1.TIF]

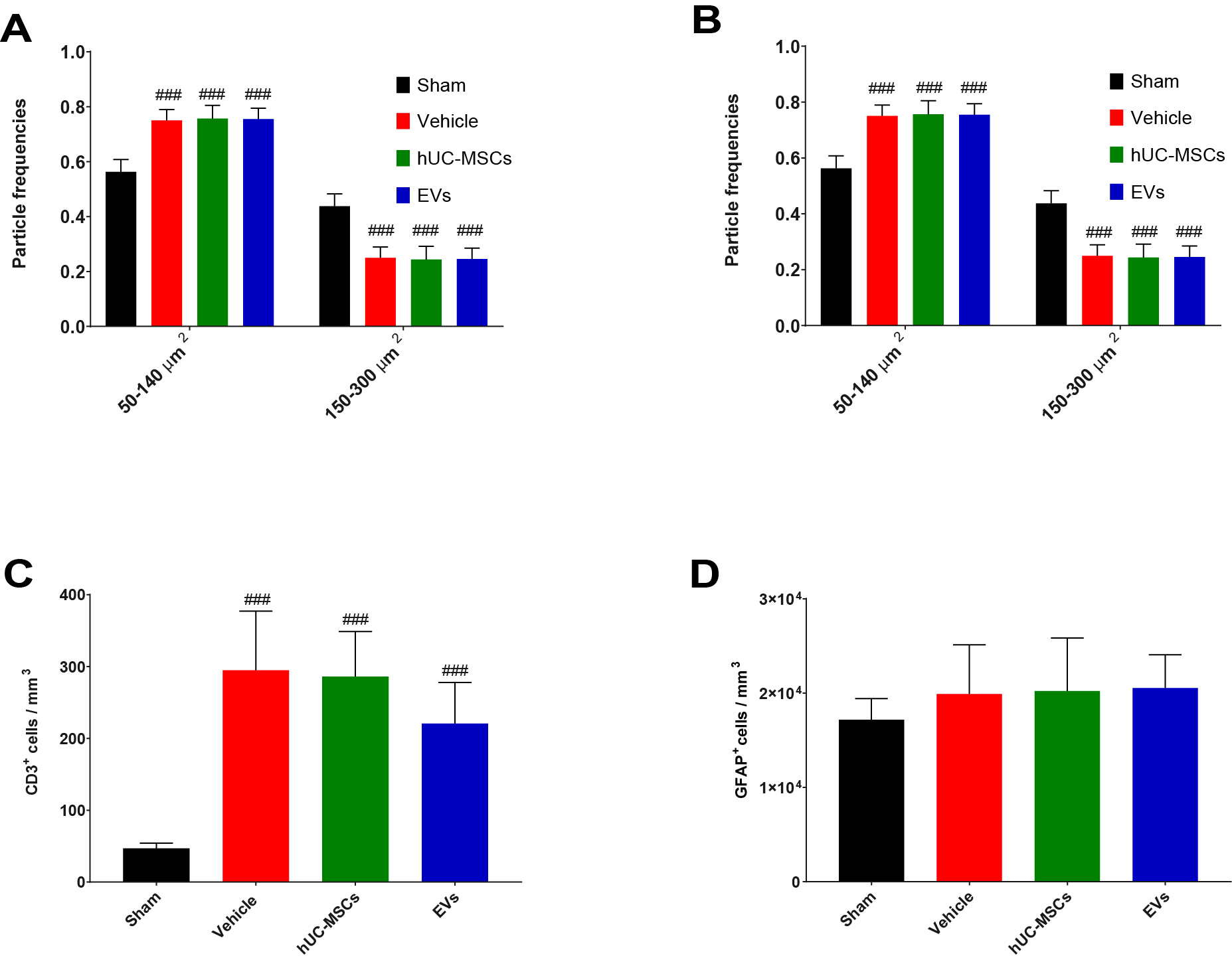

Supplement: Supplementary file 3 [file Image_2.TIF]
